# Optimizing Strength and Post-Peak Ductility in Sustainable Concretes: The Synergy of Silica Fume and Nano-Silica with Class F Fly Ash

**DOI:** 10.3390/ma19132773

**Published:** 2026-06-30

**Authors:** Grzegorz Ludwik Golewski

**Affiliations:** Department of Structural Engineering, Faculty of Civil Engineering and Architecture, Lublin University of Technology, Nadbystrzycka 40 str, 20-618 Lublin, Poland; g.golewski@pollub.pl; Tel.: +48-81-5384394; Fax: +48-81-5384390

**Keywords:** sustainable concretes, nano-silica (NS), silica fume (SF), Class F fly ash (FA), two-parameter fracture model (TPFM), fracture energy distribution, characteristic length, brittleness mitigation

## Abstract

The modification of cementitious binders using active mineral additives and nano-components represents a crucial pathway for developing high-performance, sustainable concrete composites. Nevertheless, unilateral modification of the matrix with highly reactive siliceous materials often leads to an undesirable increase in composite brittleness. This study investigates the synergistic effect of the concurrent application of nano-silica (NS), silica fume (SF), and Class F fly ash (FA) in ternary and quaternary binders, aimed at optimizing both load-bearing capacity and fracture toughness. The experimental program was conducted on seven concrete series, evaluating their mechanical parameters and non-linear fracture properties using the two-parameter fracture model (TPFM) on notched beams subjected to three-point bending. Additionally, a high-resolution energy partitioning framework was applied, decomposing the total fracture energy into four distinct components—fracture initiation energy in the elastic range (*G*_ini_), pre-peak microcracking energy (*G*_pre_), main material softening energy (*G*_soft_), and residual tail energy dissipated at large crack openings (*G*_tail_)—along with the determination of the characteristic length (*l*_ch_). The results demonstrated that while purely siliceous systems (modified with NS and SF) generate high strength increments, they simultaneously trigger a “brittleness trap,” manifested by a 13.65% decrease in the *l*_ch_ parameter. The introduction of FA effectively mitigates this hazard, transforming the failure mode into a quasi-ductile behavior. The concrete series modified with the NS+FA hybrid (Mix-5) exhibited a spectacular 107% increase in *G*_f_ and an increase in *l*_ch_ of nearly 50%, while maintaining high fracture toughness. Energy decomposition analysis in quaternary concretes confirmed a desirable reduction in the initiation energy share in favor of the softening and tail phases (*G*_tail_ reaching a record 13.1% for Mix-7), suggesting the probable activation of macroscopic crack-bridging mechanisms driven by the delayed hydration of FA particles. The research indicates that precise design of multi-component binders allows for achieving an optimal technological equilibrium point—the “sweet spot”—combining high structural capacity with safe material ductility.

## 1. Introduction

Modern concrete technology faces a dual challenge: the necessity of continuously enhancing the mechanical and durability performance of structural materials, while radically reducing the carbon footprint generated by the cement industry [1,2,3]. Portland cement (OPC) production accounts for a significant share of global CO_2_ emissions, ranging from 5% to 8% [4,5,6,7,8]. This situation necessitates the widespread application of supplementary cementitious materials (SCMs) in concrete technology, which are frequently industrial by-products from other sectors [9,10,11,12,13,14,15,16,17]. Furthermore, in current engineering practice, traditional concretes modified with a single mineral additive are giving way to advanced composites based on multi-component binders—namely ternary, quaternary, or even quinary systems [18,19,20,21]. Such an approach enables targeted tailoring of concrete properties by leveraging the distinct physicochemical characteristics, particle size distributions, and reaction kinetics of individual binder components [22,23,24,25].

The rapid development of nanotechnology has opened new horizons for modifying cementitious matrices at the atomic and molecular levels [26,27]. Among nanoadditives, nano-silica (NS) has attracted particular research interest due to its extremely high specific surface area and unique pozzolanic reactivity. Within the paste structure, NS acts as a highly active nucleation site [28,29] and effectively densifies the porous structure of the matrix, transforming large capillary pores into fine gel pores [30,31]. A crucial outcome of NS incorporation is the chemical and physical reinforcement of the Interfacial Transition Zone (ITZ) between the cement paste and the aggregate, directly resulting in substantial leaps in both early and ultimate compressive and tensile strengths of the composite [32,33,34,35].

Despite the undeniable advantages regarding the improvement in basic mechanical parameters, intensive, unilateral modification of concrete with highly reactive micro- and nano-siliceous materials (NS and silica fume—SF) carries serious structural risks [36,37], for instance the phenomenon referred to as “brittleness trap”. Composites exhibiting this characteristic behave as highly elastic materials that undergo sudden, catastrophic failure immediately upon reaching the critical load. From a structural safety perspective, the absence of a distinct post-peak deformation phase (known as softening) and a low tolerance for stress concentration around existing microdefects constitute a severe serviceability drawback, limiting plasticity and the capacity of elements to redistribute stresses [38,39,40,41].

An effective solution to the problem of excessive brittleness in concretes is the application of binder hybridization, which combines materials with starkly different reaction dynamics within the cement paste structure [42,43,44,45]. In this context, Class F fly ash (FA) serves as an excellent “partner” for the rapidly reacting NS and SF. Due to its specific spherical particle morphology and slower, time-deferred pozzolanic reaction kinetics, FA acts as an outstanding structural and energetic buffer [46,47,48]. The presence of unreacted FA grains during the initial stage inside the dense matrix generates unique macroscopic mechanisms of crack closing and bridging (i.e., crack-bridging). This multi-scale synergy (nano-NS, micro-SF, and macro-FA) allows for controlling damage evolution at every loading stage of the structural element, effectively converting brittle fracture into safe, quasi-ductile failure [49,50,51].

Traditional approaches based exclusively on classical strength tests fail to provide a comprehensive understanding of the complex phenomena occurring within the Fracture Process Zone (FPZ) of modern multi-component concretes. To objectively evaluate a composite’s resistance to crack propagation, it is essential to implement non-linear fracture mechanics tools [52,53,54]. One of the most widely recognized and specimen-geometry-independent approaches is the two-parameter fracture model (TPFM) proposed by Shah and co-workers, which enables precise determination of the critical stress intensity factor ($K_{Ic}^{S}$), critical crack tip opening displacement (*CTOD*_c_), and effective critical crack length (*a*_c_) [55,56]. A key, modern complement to this analysis is the energetic approach based on determining the total fracture energy (*G*_f_) and its detailed decomposition into components corresponding to successive material degradation phases, with a particular emphasis on the tail-phase energy (*G*_tail_). The indicator that binds all these characteristics into a macroscopic measure of fracture susceptibility is the characteristic length of the material (*l*_ch_) [57,58,59,60].

Based on the conducted literature review on this topic, it was established that research focusing on *F–CMOD* curve analysis in concretes—incorporating a detailed breakdown of individual *G*_f_ components—has been conducted only incidentally. Additionally, only a limited number of articles analyzing complex fracture processes in concretes based on multi-component binders were encountered. Previous methodological studies evaluated the influence of the loading type on the *F–CMOD* relationship [61,62,63]. From a material perspective, investigations have determined

- The effect of coarse aggregate type on the non-linear crack propagation process in ordinary [64] and high-performance concretes [65];
- The combined effect of binary binders on the *F–CMOD* relationship and total *G*_f_ variations resulting from material modifications, namely SF and FA [66,67,68]; SF, FA, and polypropylene fibers [69]; as well as SF and NS [70].

Considering the identified research gap in describing the post-peak behavior of concretes made with hybrid binders, the primary objective of this work is a comprehensive assessment of the synergistic effect of NS, SF, and FA in ternary and quaternary binders on the non-linear fracture mechanics characteristics of modern structural concretes [71,72,73,74]. The scope of research involves laboratory three-point bending tests of notched beams for seven distinct concrete formulations (ranging from the reference mix based solely on OPC to advanced quaternary systems). Based on the obtained experimental *F–CMOD* curves, the specific objectives of the work are

- To determine the key parameters of the TPFM;
- To conduct an innovative analysis of the fracture energy component distribution at individual stages of crack propagation;
- To establish the sensitivity of the *l*_ch_ index to the implemented material modifications.

These analyses aim to precisely pinpoint the technological equilibrium point—the “sweet spot”—that bridges high load-bearing capacity with the desired level of material ductility.

## 2. Materials and Experimental Methods

### 2.1. Materials

#### 2.1.1. Rationale for Material Selection and Concept of Cementitious Binder Modification

In the presented research, the concept of modifying the cementitious binder with pozzolanically active mineral additives and nanoadditives was utilized to evaluate the specific characteristics of the composites during the fracture process under external loads. Through these adjustments, it is possible to tailor concrete properties to specific technical, economic, and environmental requirements compared to composites based solely on ordinary Portland cement. In modern engineering practice, structural concretes very frequently rely on multi-component binders, as they allow for the “targeted” engineering of material properties for specific applications. Therefore, three distinct groups of concretes were used in this study, namely those in which OPC was replaced by one, two, or three admixtures characterized by both fine particle sizes and high specific surface areas. This modification aimed to improve critical fracture mechanic parameters of the analyzed concretes and enhance the capability of the composites to transmit micro-damage through the damaged material structure, thereby minimizing the risk of sudden and uncontrolled failure of the structural element [24,75,76,77,78].

Based on the findings of previous studies in this field, FA, SF, and NS were selected to prepare the concrete mixtures. The percentage ranges of individual materials used as OPC substitutes corresponded to their optimal amounts utilized in typical structural concretes. Based on these data, this study adopted specific replacement levels of the primary binder with various combinations of FA, SF, and NS. This procedure was designed to elicit a synergistic effect among the combined admixtures, which was subsequently evaluated regarding the impact of the proposed material modifications on altering the damage initiation and propagation processes in cement concretes [79,80].

In the presented research, the methodology for determining the composition of the concrete mixtures involved replacing the cementitious binder in the concrete mix in amounts of 0%, 5%, 15%, 20%, and 30%. When designing the concrete mix proportions, constant replacement levels of OPC by SF at 10% and NS at 5% by weight of OPC were established as effective boundary values for applying these admixtures within concrete structures. The specific replacement levels for SF and NS were selected based on widely recognized literature guidelines. Previous extensive studies have demonstrated that the optimal dosage for enhancing both mechanical properties and durability typically ranges from 5% to 10% by mass for SF [81,82] and between 1% and 3% by mass for NS in binary cement systems, as higher concentrations of nanoparticles may lead to agglomeration issues and a decrease in concrete performance [41,83]. On the other hand, higher NS dosages of up to 5% by mass have been proven highly effective in multi-component binder systems containing additional SCMs, such as FA and SF. In such complex matrices, synergistic effects—specifically enhanced hydration and significant matrix densification—justify the increased nano-silica content [84,85].

Additionally, the inclusion of these two materials in the concrete mix composition significantly seals the cement matrix structure and substantially accelerates hydration processes, the measurable effect of which is the reinforcement of the ITZ between the aggregate grains and the cement matrix. This mechanism also alters the destructive processes of concretes featuring such binder modifications. The boundary replacement level of OPC by the primary SCM, namely FA, was set at up to 30% by weight of the cement, representing the upper limit for utilizing this industrial waste in low-volume FA concrete [86,87,88,89]. The specific variable dosages of FA (i.e., 5%, 15%, and 25%) were strategically selected based on two primary criteria. First, these replacement levels were precisely designed to ensure that the cumulative content of all mineral additives in the multi-component binder systems (ternary and quaternary) reaches exact benchmarks of 20% and 30% by mass, which represent common thresholds in industrial concrete technology. Second, this formulation allows for a direct and reliable comparison of the current synergistic matrices with the author’s previous baseline studies, where traditional concrete series modified solely with fly ash at equivalent total replacement levels of 20% and 30% were extensively evaluated. The detailed properties of the SCMs and other materials used to prepare the concrete mixtures are provided in Section 2.1.2, while the exact proportions of all components for each concrete series are summarized in Section 2.2.

#### 2.1.2. Material Properties

The constituting materials of concrete used in the present study are natural gravel as coarse aggregates [90] (with specific gravity 2.65 g/cm^3^ and aggregate size 2.0–8.0 mm) and natural sand as fine aggregates (specific gravity of 2.60 g/cm^3^ and maximum size of 2.0 mm) according to DIN 4226-1:2001-07 [91] and EN 13791:2019-12 [92]. In addition, the main binder and the supplementary cementitious materials (SCMs), which were used as partial replacement of OPC to produce binary, ternary and quaternary mixes, included

- Ordinary Portland cement (OPC) CEM I 32.5 R according to EN 197-1:2011 [93], produced by the Chełm cement plant (Chełm, Poland);
- Class F FA, according to ASTM C 618-03 [94], produced by Puławy thermal-electric power station (Puławy, Poland);
- Non-condensed SF, obtained from Łaziska Ironworks (Łaziska Górne, Poland);
- NS Konasil K-200, produced by the OCI Company Ltd. located in Seoul (Republic of Korea).

The detailed chemical composition and physical properties of the binders used in the study are summarized in Table 1 and Table 2, respectively.

The chemical composition of both additives was determined by the XRF method. A Epsilon 3X spectrometer was applied (Malvern Panalytical, Malvern, UK). On the other hand, analyzed physical parameters were assessed as follows:

- Specific density by pycnometric method;
- Specific surface area according to the Blaine method;
- Particle size distribution by laser granulometry using measuring device Masterizer 3000 and measuring range 0.01–3500 μm (Malvern Panalytical, Malvern, UK);
- Loss of ignition by burning the individual materials for one hour at 1000 °C;
- Color—visually.

To produce concrete mixtures, liquids were also required, i.e.,

- Superplasticizer (SP) STACHEMENT 2750 based on polycarboxylates (1.8% of binding material weight);
- Domestic water supply that meets the requirements of EN 1008:2002 [95].

**Figure 1:**
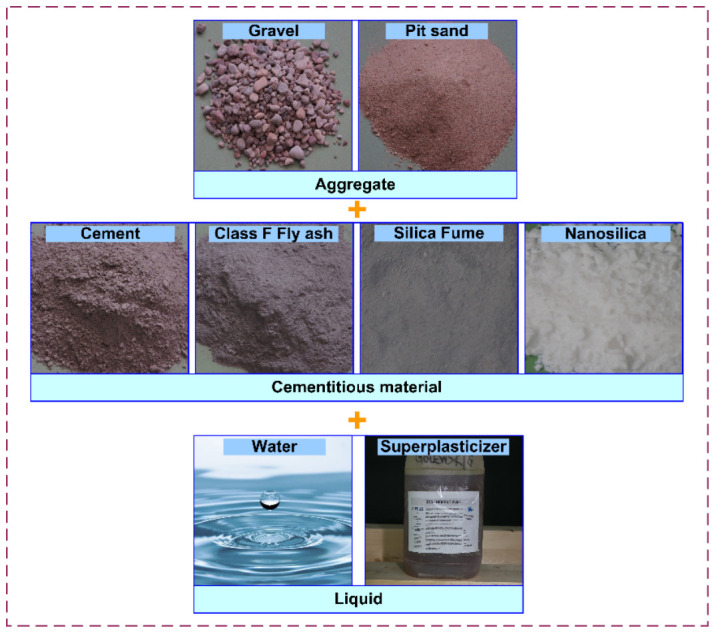
The raw materials.

The appearance of all materials used in this study is shown in Figure 1.

### 2.2. Mix Proportions

In order to assess the strength, as well as brittleness, ductility, and fracture processes of concretes, including combinations of the SCMs, six concrete mixes containing a modified binder including OPC, FA, SF and NS, and a reference mix containing 100% cementitious binder were manufactured. Cement paste was prepared with a water/binder ratio of 0.4. Six specimens were prepared for each group. Table 3 presents the mix proportions of the concrete compositions, with specifications of the percentage contents of individual binders in each concrete mix. It should be explicitly noted that in all modified series, the OPC was partially replaced by individual types of SCMs by mass.

### 2.3. Specimen Fabrications

**Figure 2:**
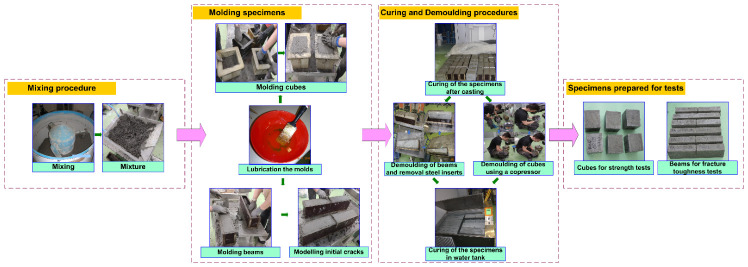
Specimen preparation procedure.

The complete process of specimen preparation for testing is illustrated in Figure 2. The mixtures were prepared in a forced-action counter-current mixer. First, the dry components were mixed in the following order: fine aggregate with coarse aggregate, followed by OPC, FA, and SF. This stage lasted for a total of 5 min, during which both types of aggregates were mixed together for 2 min, and for the subsequent 3 min, the aggregates were mixed with the cementitious materials. Following this procedure, half of the water content required for the mixtures was added and blended with the NS and SP. This process continued for another 2 min and was aimed at preventing the agglomeration of the extremely fine NS particles. The use of an efficient SP allowed for their precise dispersion within the structure of the concrete mixture. In the final stage, the mixture was supplemented with the remaining portion of water. Subsequently, all components were mixed for an additional 2–3 min until a homogeneous, properly consolidated concrete mixture was achieved.

Specimens for mechanical strength testing were prepared in plastic molds in accordance with the EN 12390-2:2019 standard [96], while beams for fracture toughness testing were prepared in bolted wooden molds (Figure 2). Before placing the concrete mixture into the molds, they were thoroughly coated with a release agent, as shown in Figure 2. Cubic specimens with a side length of 150 mm and beams with dimensions of 80 × 150 × 700 mm were cast. The mixture was placed in the molds in several layers. After placing each layer, the mixture was compacted on a vibrating table. The final layers were carefully smoothed using finishing trowels. In the case of the beams, after placing the concrete mixture, steel inserts for forming the initial notches were positioned at mid-span during the final stage (Figure 2).

After fabrication, the specimens were successively sprinkled with water and covered with a layer of polyethylene film to ensure optimal curing conditions. After 24 h, the specimens were demolded. Cubes cast in plastic molds were demolded using an air compressor, whereas the beams were demolded starting with the removal of the steel inserts, after which the molds were unbolted, thereby releasing the fabricated beams. The demolding process of the specimens was carried out in multiple stages, as illustrated in Figure 2. After demolding, the specimens were stored in a water curing tank at a temperature of 20 ± 2 °C and a relative humidity of RH = 95–100% for a period of 14 days. Subsequently, for the next 2 weeks, the specimens were cured under laboratory conditions with the prevailing thermal and moisture conditions of t = 20 ± 2 °C and RH = 40%. A view of the specimens prepared for strength and fracture toughness testing after the full casting and curing process is shown in Figure 2.

### 2.4. Testing Apparatus and Methods

#### 2.4.1. Mechanical Parameter Testing

The experiments evaluating the strength parameters of concrete composites incorporating FA, SF, and NS were carried out under static loading. The following loading rates of the specimens were adopted:

- 0.5–0.8 MPa/s—during the compressive strength test;
- 0.06–0.04 MPa/s—during the splitting tensile strength test.

The testing of the main strength characteristics of the analyzed composites, i.e., *f*_cm_ and *f*_ctm_, was carried out on testing machine (Walter + Bai ag, type NS19/PA1; Löhningen, Switzerland) with a maximum capacity of 3000 kN. Six specimens were prepared for each mixture ratio and for both mechanical tests. The following standards were applied to conduct the strength tests:

- EN 12390-3: 2011 + AC: 2012 [97]—for the compressive strength test;
- EN 12390-6: 2009 [98]—for the splitting tensile strength test.

**Figure 3:**
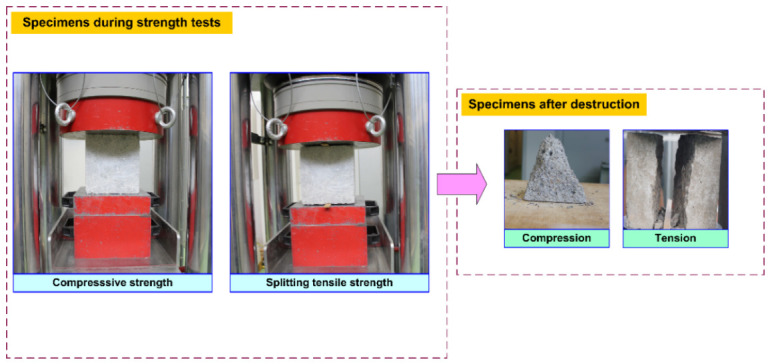
Specimens during strength tests and after failure.

A view of the specimens during both types of strength tests and after failure, as a result of the experiments, is shown in Figure 3.

#### 2.4.2. Linear Elastic Fracture Mechanic Parameter Evaluation

**Figure 4:**
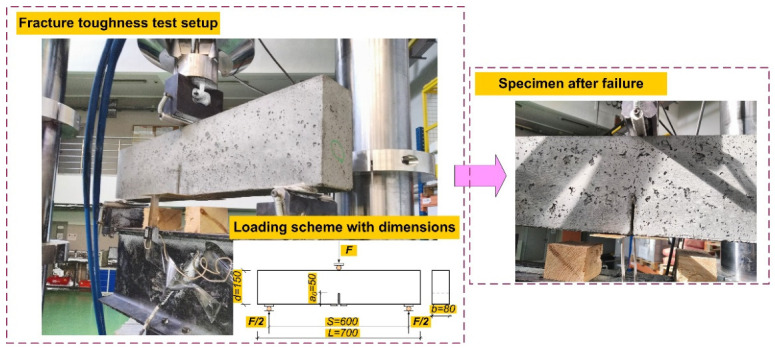
Test stand of fracture toughness tests and a view of specimen failure.

Fracture toughness tests were conducted according to the RILEM draft recommendations TC-89 FMT [99] using an MTS 810 testing machine (Eden Prairie, MN, USA). The notched beams had dimensions of d = 150 mm, b = 80 mm, L = 700 mm, S = 600 mm, and a_0_ = 50 mm (Figure 4).

The tests were performed under crack mouth opening displacement (CMOD) control using an axial MTS clip gage 632.03F-3 extensometer (Eden Prairie, MN, USA) mounted on attached knife edges. The loading rate was adjusted so that the maximum load was reached in approximately 5 min. Unloading to 0 kN and subsequent reloading cycles were performed at roughly 95% of the post-peak load to record stable F–CMOD curves [100].

The resulting *F–CMOD* envelope curves filter out hysteresis noise, providing a smooth, representative post-peak softening curve. This enables precise calculation of the total fracture energy (G_f_) and accurate assessment of the structural load-bearing capacity throughout all failure stages, including the final tail phase prior to complete failure.

**Figure 5:**
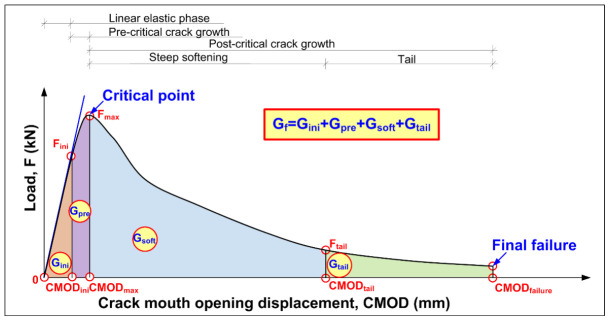
Methodology for partitioning the total fracture energy *G*_f_ into characteristic components: *G*_ini_, *G*_pre_, *G*_soft_, and *G*_tail_.

As illustrated in Figure 5, the methodology for partitioning the total fracture energy (*G*_f_) isolated four components corresponding to the specific crack propagation stages evaluated in this study:

- *G*_ini_—fracture initiation energy (elastic range);
- *G*_pre_—pre-peak phase energy (microcracking);
- *G*_soft_—main material softening phase;
- *G*_tail_—energy dissipated in the final fracture phase at large crack openings.

The partitioning of the *F–CMOD* graph (as presented in Figure 5), particularly the definition of the post-peak parameters *F*_tail_ and *CMOD*_tail_, was introduced to accurately evaluate the total fracture energy. Due to the asymptotic nature of the post-peak softening behavior in concrete, continuing the test until the applied load reaches absolute zero is technically difficult and impractical. Therefore, the test was intentionally terminated at *CMOD*_tail_, corresponding to the residual load *F*_tail_. In this study, this cut-off point was defined as the moment when the load dropped to approximately 5% of the *F*_max_ or when the *CMOD* reached a limiting value of 3.0 mm. Partitioning the graph in this manner allows for a precise calculation of the measured work of fracture up to *CMOD*_tail_ and provides a mathematical basis for estimating the unrecorded tail area using analytical approximations, thereby preventing the underestimation of the total fracture energy.

To clarify the novelty of the applied fracture energy decomposition approach, it is important to contrast it with conventional literature methods. Traditional fracture mechanic frameworks (such as the standard RILEM or Hillerborg methods) typically evaluate the concrete brittleness through a single, global value of *G*_f_, or at most, a binary pre-peak/post-peak simplification. While useful, such macro-evaluations mask the distinct sub-stages of internal structural degradation. The novelty of the proposed approach lies in its high-resolution, four-component energy partitioning (*G*_ini_, *G*_pre_, *G*_soft_, and G_tail_). By isolating the energy specifically dissipated during stable microcracking (*G*_pre_), major unstable macro-crack propagation (*G*_soft_), and the final aggregate bridging/interlocking phase (*G*_tail_), this method provides an unprecedented analytical resolution. This allows for a precise, quantitative mapping of how different combinations of SCMs (NS, SF, and FA) actively alter specific physical phenomena at distinct operational timelines of the failure process, rather than just recording the final macroscopic collapse.

It should be noted here that to maintain clarity, in the subsequent part of this paper (see Section 3), color consistency of the charts with the model presented in Figure 5 was preserved.

## 3. Research Results and Discussion

### 3.1. Determination of Strength and Fracture Mechanic Parameters

To ensure the clarity of the presentation and to provide a comprehensive summary of the analytical methods used, all formulas for calculating strength and fracture mechanic parameters are summarized in Table 4. The table was divided into three sections, each containing a base of equations regarding a specific group of analyzed parameters, namely,

- Basic strength properties (parameters from group A);
- Fracture mechanic parameters as part of the two-parameter fracture model (TPFM) (parameters from group B);
- Energetic and brittleness parameters (parameters from group C).

Table 4 included the symbol, the parameter name, and the complete path of equations and sub-equations used to determine the individual analyzed material parameters.

### 3.2. Methodology of Experimental Results Presentation

The mean results obtained from the experimental tests, subsequently calculated using the formulas summarized in Table 4, are presented in bar charts. To precisely illustrate the effect of individual modifiers on the mechanical properties and fracture toughness of the concrete, a unified data presentation system was applied across all bar charts. In addition to the mean values of individual parameters, displayed at mid-height of the bars, error bars (standard deviations) representing the scatter of results were plotted. Furthermore, to facilitate a comparative analysis, the percentage change in each specific property relative to the reference concrete (Mix-1) was calculated for each modified mix (from Mix-2 to Mix-7). These changes are illustrated graphically: green upward-pointing triangles (▲) denote an increase, whereas red downward-pointing triangles (▼) indicate a decrease in the value of the analyzed parameter.

Color consistency between the bar charts and the *F–CMOD* envelope curves was maintained. The color of each bar corresponded to a different concrete series and was identical to the color of the envelope curve for that material.

### 3.3. Mechanical Properties

**Figure 6:**
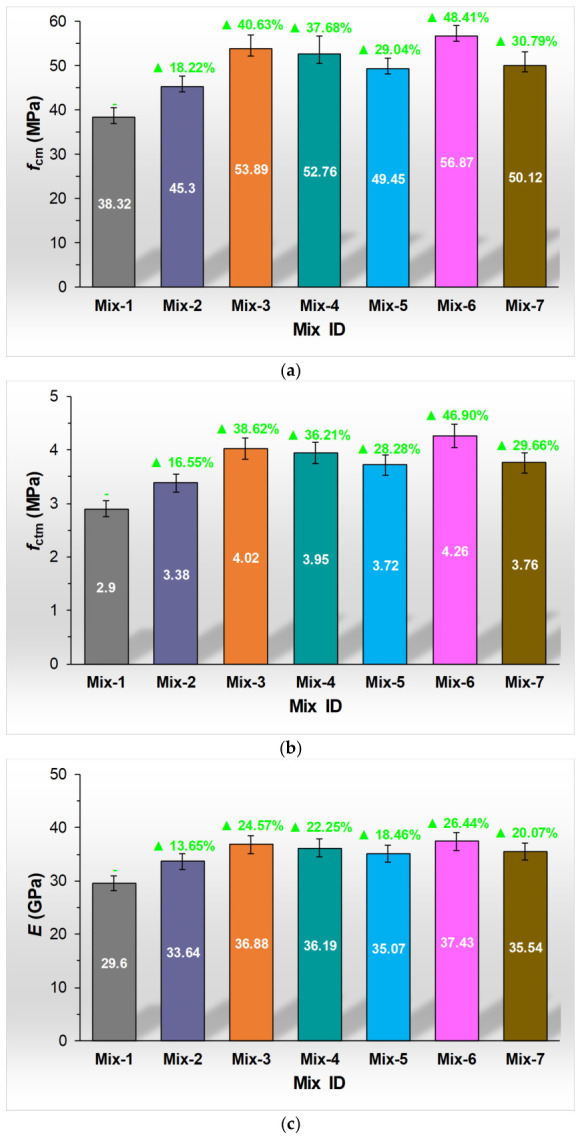
Mechanical properties of the tested concretes: (**a**) compressive strength, (**b**) splitting tensile strength, (**c**) Young’s modulus.

Figure 6 summarizes the results of the basic mechanical parameters of the analyzed concretes, i.e.,

- Compressive strength, *f*_cm_;
- Splitting tensile strength, *f*_ctm_;
- Modulus of elasticity, *E*, determined during bending.

At this point, it should be noted that while both strength parameters were determined in destructive tests according to the procedures described in Section 2.4.1, the modulus of elasticity was determined using an indirect method based on the initial compliance of the *F–CMOD* envelope curves. This procedure, based on the RILEM Draft Recommendations, TC 89-FMT [99], is described in detail in Section 3.4. The equations necessary to determine all analyzed parameters are provided in Table 4.

The test results of the mechanical parameters of the analyzed concretes shown in Figure 6 demonstrate a distinct influence of the applied cementitious binder modifications on the obtained outcomes. Relative to the reference concrete, i.e., Mix-1, all series with mineral additives achieved higher values of *f*_cm_, *f*_ctm_, and *E*.

As can be observed, the addition of NS alone in Mix-2 brought a moderate increase in both strength parameters by 18% and 16%, respectively, and an increase in *E* by over 13%. The somewhat indistinct effect of mechanical parameter improvement in this concrete series was likely due to the fact that while NS has an enormous specific surface area and acts very aggressively chemically, its independent application is associated with certain technological problems and limitations. This material significantly increases the water and superplasticizer demand, easily agglomerates, disperses poorly, and can cause excessive autogenous shrinkage. Additionally, NS primarily refines very fine pores and is unable to comprehensively densify the composite structure. The above problems limit the effectiveness of the practical use of NS in concrete, and its independent application does not yield spectacular results regarding the concrete strength parameters. For these reasons, it is recommended to combine this active nano-admixture with other SCMs to achieve a synergistic effect on the concrete structure.

Therefore, a more significant improvement in all analyzed mechanical parameters, i.e., *f*_cm_, *f*_ctm_, and *E*, was brought about by modifying the binder with two types of silica. In the case of the combined addition of SF and NS in the Mix-3 series concrete, a 40% increase in *f*_cm_ and *f*_ctm_ and a nearly 25% increase in *E* were achieved. The results obtained for the concrete with the combined addition of both siliceous materials are thus very favorable. They clearly exceed the results obtained by other researchers analyzing the properties of concrete composites modified in the same manner. Previous studies have shown that modifying the binder composition with a combined addition of NS+SF causes an increase in *f*_cm_ and *f*_ctm_ values by 17% and 23%, respectively [103]. Conversely, another study reported a 28-day increase in the *f*_cm_ parameter by 18.57% [104]. The above two articles also demonstrated that such material modification brings other beneficial consequences, causing concretes based on such binders to be characterized by increased resistance to carbonation, reduced sorptivity and water absorption, limited content of harmful pores, higher sulfate resistance, and higher abrasion resistance [105,106]. Moreover, the mechanical parameter values in the Mix-3 series concrete exhibited the second-highest result among the entire set of analyzed composites. The compressive strength of this concrete was almost 54 MPa, the tensile strength was over 4 MPa, and the modulus of elasticity was nearly 37 GPa (Figure 6).

It should be noted that such favorable mechanical parameter results obtained for the concrete containing two types of pozzolanically active siliceous materials are the result of their mutual multi-scale interaction. The NS grains themselves are primarily active locally within the concrete structure and strongly accelerate the early hydration of cement. Additionally, due to the very high specific surface area of this material, as mentioned above, NS particles easily form agglomerates. Therefore, often, not all NS grains are able to effectively participate in the process of forming additional hydration products. For these reasons, the addition of NS alone is not as effective as its combination with active SF.

The synergistic effect of both mineral admixtures has been proven, among others, in studies [105]. It is related to the fact that both materials act at different levels of the microstructure scale and complement each other. When both types of silica are combined, the effect of multi-scale particle packing occurs. This phenomenon is manifested by SF filling the pores and spaces between cement grains at the micrometer scale, while nano-silica fills even smaller spaces at the nanometer scale. This results in concrete characterized by

- Lower porosity;
- A denser cement paste structure;
- A better ITZ around the aggregate grains.

Nano-silica is applied to concrete in very small amounts, i.e., up to 5% by weight of cement. Therefore, being alone in the concrete composite structure, it reacts rapidly and does not provide a long-term reinforcement effect [107]. Conversely, SF is typically added in an amount of 10% or more. This ensures a long-term effect of its grains’ interaction. In turn, the coexistence of SF and NS means that a rapid increase in material strength from the participation of very fine NS particles can be observed in the concrete structure, followed by further densification and strength increases resulting from the presence of fine SF grains [106]. The presence of SF improves the dispersion of NS nanoparticles and stabilizes the overall particle system of the cementitious composite. As a result, a larger portion of NS actively participates in pozzolanic reactions [108]. This leads to the formation of a more homogeneous composite microstructure and a distinct reduction in harmful pores. Additionally, the SF+NS system also improves the ITZ structure [109]. The collaboration of both materials affects this concrete zone by

- Reducing the amount of large portlandite crystals;
- Increasing the amount of C-S-H gel;
- Decreasing the number and size of microcracks.

The combined interaction of both siliceous additives ensures that, together with OPC, they create a much more compact cement matrix structure in the concrete than if each of these materials were used separately. Consequently, this leads to a distinct improvement in the mechanical and strength parameters of concretes with the combined addition of SF+NS compared to concretes containing only NS (Figure 6). Given that replacing OPC with siliceous additives also affects the densification of the cement paste structure in the ITZ area and the reduction in cracking levels in this area, it can also be presumed that the concrete of this series will be characterized by favorable fracture mechanic parameters.

Another group of materials analyzed in the present study consisted of ternary concretes based on the combined incorporation of NS and FA admixtures, namely the Mix-4 and Mix-5 series. Their example illustrates the influence of FA on altering the strength parameters in concretes based on the synergy of cementitious binder components. Comparing the mechanical parameter values obtained for the Mix-4 and Mix-5 series concretes, it is evident that increasing the FA content while keeping the NS proportion constant leads to a slight decrease in *f*_cm_, *f*_ctm_, and *E* compared to the series with the highest parameters. Nevertheless, the results obtained for these concretes still remain significantly higher than those of the reference concrete (Figure 6).

However, the highest strength parameters by far were achieved by concretes with the greatest diversification of the binder composition, i.e., those based on quaternary binders. The Mix-6 mixture, containing a small 5% amount of FA in addition to NS and SF, exhibited the highest compressive strength (56.87 MPa) and tensile strength (4.26 MPa), as well as the highest stiffness (E = 37.43 GPa). This phenomenon may be attributed to the strong synergistic interaction of all three mineral admixtures at different levels of the cement paste structure, which, as is consistent with previous studies, effectively densifies the concrete composite structure. Additionally, the favorable effect of a distinct increase in mechanical parameters in this concrete simultaneously results from

- The difference in particle sizes;
- Different pozzolanic reactivities;
- Diverse hydration kinetics;
- Improved particle packing;
- Modification of the ITZ microstructure to an even greater extent than in binary and ternary binders;
- Capillary porosity reduction.

Due to the distinct differences in grain size among the individual binder types, the actual mechanism of their mutual interaction is that each mineral admixture densifies different zones within the cement matrix structure. FA fills the spaces between cement grains, SF fills the micro-spaces, while NS seals the nanopores.

Additionally, because each mineral admixture reacts at a different rate and its period of highest pozzolanic activity falls at a different time of cement paste curing, the development of the C-S-H phase can be prolonged and more continuous. NS reacts very quickly and reinforces the material structure after just 1–3 days. SF reacts relatively fast and develops medium-term strength. In this regard, FA reacts the slowest of all three SCMs, but it is capable of increasing strength 28–90 days after mixing.

Another significant interaction mechanism between NS, SF, and FA grains is the so-called kinetic synergy. It involves the highly reactive NS accelerating cement hydration and creating additional C-S-H nucleation sites on its grains. Consequently, this also improves the activation of SF and FA. As a result, FA practically becomes more reactive in the presence of NS than when it appears in the paste alone or with other SCMs. This phenomenon has been confirmed in microstructural studies of concretes containing NS and FA [110,111].

The remaining three mechanisms listed above, which significantly and positively affect the concrete structure in the presence of the combined addition of NS, SF, and FA, are somewhat interconnected and mutually interactive. The reorganization of the distribution of pozzolanic micro-fillers leads to the densification of both the cement matrix structure and the ITZ area of the aggregate grains. This, in turn, causes a reduction in capillary porosity in the condensed paste structure and a reduction in microcracks in the reinforced ITZ. As a consequence of all the interaction mechanisms discussed above between NS, SF, and FA, the benefits of the combined use of these SCMs in concrete are based on the three following effects of their mutual interaction, i.e.,

- Synergistic effect;
- Multi-scale densification;
- Optimized particle packing.

Their occurrence makes it possible to obtain concretes with lower porosity, higher structural density, and consequently significantly improved mechanical and strength parameters (Figure 6).

### 3.4. Analysis of F–CMOD Envelope Curves

**Figure 7:**
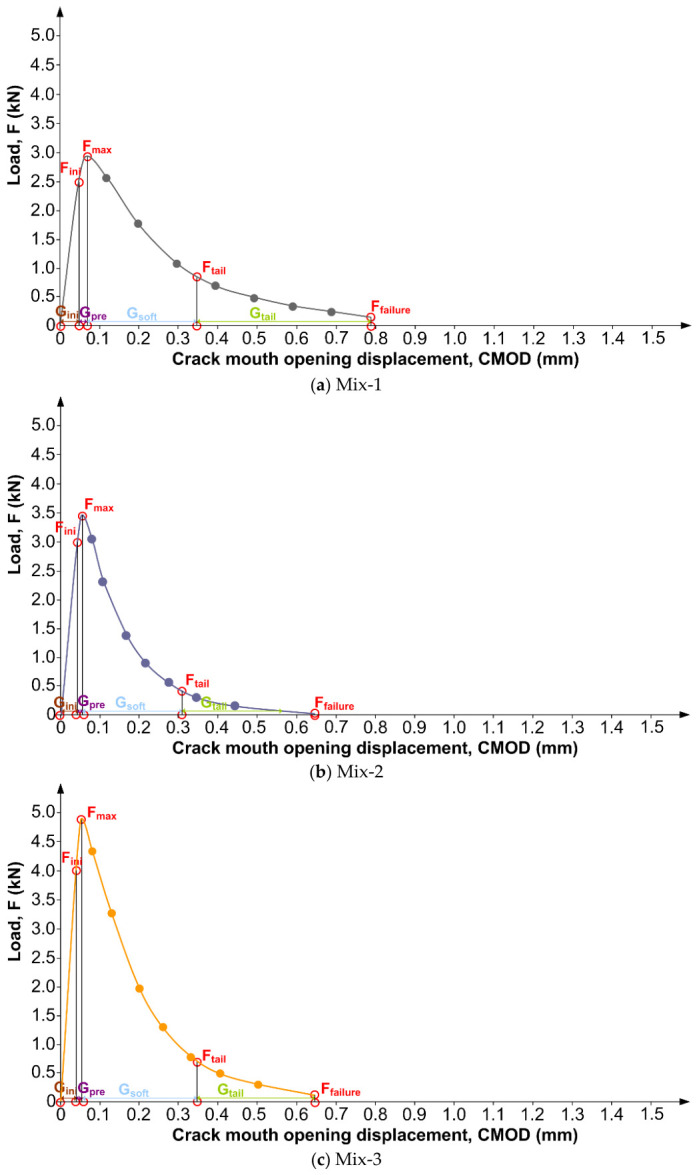

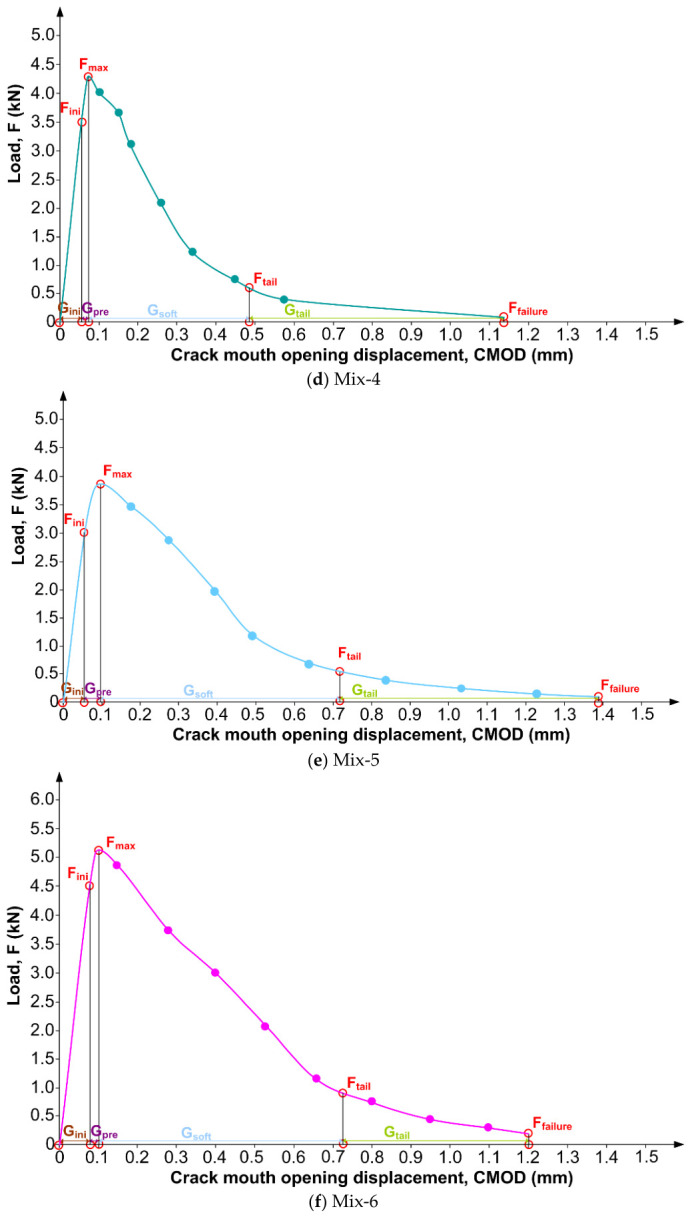

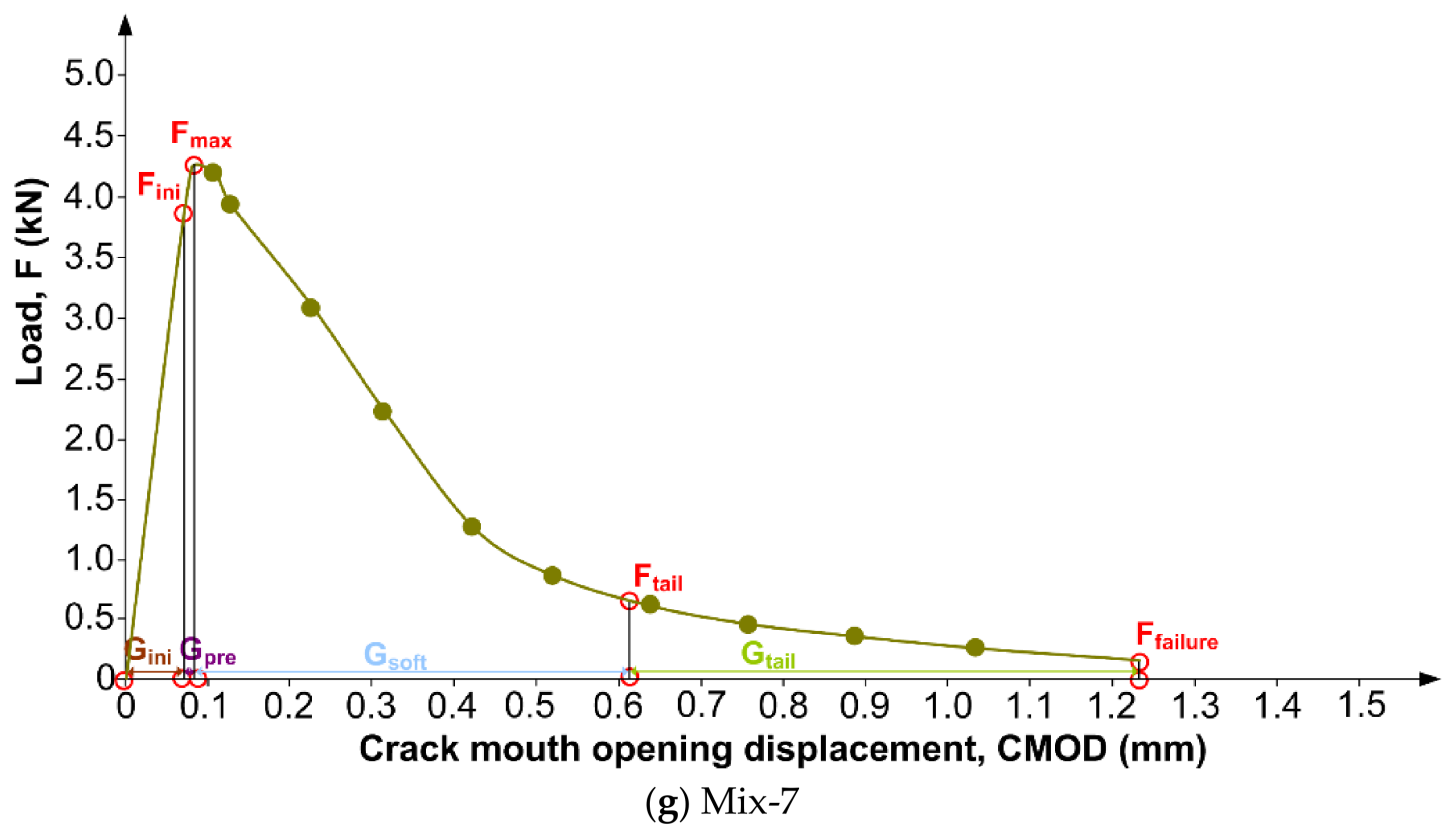
Experimental *F*–*CMOD* envelope curves illustrating the full fracture process for all mixes.

The full picture of the failure phenomenon of all concrete series was recorded on the *F–CMOD* envelope curves. Their representative images, generated according to the methodology presented in Figure 5, are shown in Figure 7.

These graphs mark and describe all characteristic points that are part of the *F–CMOD* relationship, i.e., the locations of forces at various stages of the specimen’s damage process and the ranges of the individually selected fracture energies. Based on the developed *F–CMOD* envelope curves, all significant parameters directly related to the crack propagation process and the material’s failure rate were also specified for further analysis for each of the tested concretes. The equations used to determine these parameters are given in Table 4, while their average values obtained from the calculations are presented in Table 5.

Analyzing the *F–CMOD* curves shown in Figure 7, it is clearly evident that the envelope curves for mixtures with a dominant share of silica fume (especially Mix-3) exhibit a sharp peak and a sudden drop in load in the softening zone (Figure 7b,c). Such an image of crack development in the initial phase of crack growth indicates a high brittleness of the material and a tendency for rapid release of elastic energy after exceeding the critical stress. Confirming the brittle behavior of concretes with the addition of siliceous materials is the lowest values of both *CMOD*_max_ and the compliance ratio *C*_u_/*C*_i_, which were observed for the Mix-2 and Mix-3 series concretes (Table 5). Both the shape of the curves obtained for these concretes and the analyzed parameters listed in Table 5 unambiguously demonstrate that these are materials with a low capacity for stress redistribution, minor deformability before failure, and a rapid transition from crack initiation to unstable crack propagation. The analysis of the *F–CMOD* curves in the Mix-2 and Mix-3 series concretes indicates that microcracks in their structure developed briefly, and the material had no tendency to work after the appearance of the first crack. Upon the occurrence of damage, the concretes underwent sudden softening and a rapid transition into the softening region. This was followed by an almost negligible tail phase, and the specimen rapidly underwent complete destruction (Figure 7b,c).

Conversely, the addition of FA in mixtures from Mix-4 to Mix-7 alters the character of the failure curves. The *F–CMOD* curves become wider and exhibit characteristic local disturbances in the post-peak zone, consisting of sudden, minor drops and recoveries of the sustained load. This phenomenon, thoroughly analyzed in a previous study [112], may be associated with multi-stage cracking (crack branching) and delayed hydration of FA grains. This effect is most pronounced in the concrete with the highest FA content, i.e., the Mix-5 series. However, in the Mix-7 series concrete, where the FA content was optimized using other admixtures, i.e., NS and SF, this phenomenon is slightly “calmed”. It should be noted that all concretes with the addition of FA are characterized by a clearly longer tail phase compared to the siliceous concretes, i.e., the Mix-2 and Mix-3 series (Figure 7). Significantly higher *C*_u_/*C*_i_ values were also observed in these concretes, especially for the series with the highest FA content, i.e., Mix-5 and Mix-7 (Table 5). Both the failure curve pattern and the compliance level of these materials in the damage phase testify to the material’s ability to carry loads at large crack openings. Such behavior, in turn, is typical of concretes with enhanced ductility, in which the material, after reaching the maximum force, can still transfer stresses despite the increasing crack opening.

Based on the graphs shown in Figure 7 and the data contained in Table 5, it can thus be concluded that a brittle and very brittle specimen failure character was observed in concretes with the addition of NS alone and NS+SF. These concretes exhibited low stress redistribution capacity, low ductility, and sudden failure after crack initiation. The very short tail phase in these concretes indicates that they lost their load-bearing capacity almost immediately after the first cracking. In essence, these materials lacked effective mechanisms to arrest crack development and showed no crack-bridging symptoms. Consequently, in the concretes of these series, the transition from a load-bearing state to a loss of load-bearing capacity occurred very quickly.

On the other hand, in concretes with the addition of FA, a tendency towards gradual degradation of the material stiffness after cracking and stable crack propagation instead of sudden failure could be observed. Effective crack-bridging mechanisms became evident in the material. This meant that a portion of the material continued to transfer stresses, while the delayed-acting active FA grains arrested the crack development. Therefore, a successive dissipation of energy occurred during the material destruction process, leading to a stable development of cracking in the composite structure. Concretes with the addition of FA, particularly the Mix-5 and Mix-7 series, failed in a very stable manner, i.e., along a significantly prolonged path slowly leading toward complete material destruction.

The characteristic disturbances visible on the *F–CMOD* curves for concretes with the addition of FA in the softening phase result from the crack branching mechanism and FA activation, which “stitches” the crack front thanks to delayed hydration. It should be added that the phenomenon of local disturbances in the crack propagation process in FA-modified concretes was most clearly visible in the concretes with the highest amount of this waste material, i.e., the Mix-5 and Mix-7 series. It occurred primarily when the concretes of these series reached the Fmax loads and transitioned into the softening phase (Figure 7d,g).

While the present findings align with several previous studies indicating that highly reactive silica additions (NS and SF) significantly enhance the peak load and pre-peak energy dissipation (*G*_pre_) due to rapid matrix densification, a critical comparison reveals important differences regarding the post-peak behavior. For instance, several studies in the literature (82–87) have reported a distinct embrittlement and a severe drop in the post-peak fracture energy when using high volumes of SF or NS alone. This literature discrepancy is primarily attributed to the high susceptibility of such dense matrices to autogenous shrinkage and early-age microcracking. In contrast, our results demonstrate that this pronounced brittleness is fundamentally mitigated in the proposed multi-component system. We hypothesize that this discrepancy stems from the synergistic presence of FA. The slower pozzolanic reaction of FA, combined with the stress-relieving “ball-bearing” effect of its spherical particles, effectively offsets the internal stresses generated by NS and SF. Consequently, unlike the single-addition systems widely reported in the literature, our hybrid mixtures shift the failure mechanism from a sudden, brittle rupture to a more gradual and controlled energy dissipation in the softening (*G*_soft_) and tail (*G*_tail_) stages (Figure 7).

**Figure 8:**
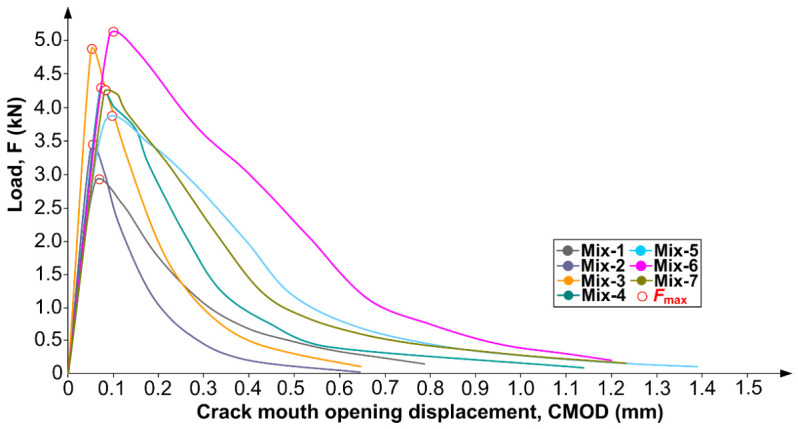
Consolidated chart of the *F–CMOD* curves for all tested mixtures.

To further support the comparative discussion and visually emphasize the differences in the mechanical response, particularly in the post-peak softening region, a consolidated plot presenting the *F–CMOD* curves for all tested mixtures is provided in Figure 8. This comprehensive representation allows for a direct observation of how the specific SCM combinations influence the actual load-bearing capacity and the subsequent energy dissipation capabilities during the crack propagation phase.

In addition, based on the results obtained from the *F–CMOD* envelope curves, an in-depth evaluation was also carried out regarding both the proportions of forces F occurring at individual stages of crack propagation in the material and the changes in crack opening displacement *CMOD* values at crucial moments of material destruction. A thorough analysis of both these parameters allowed for a precise assessment of the ductility of the material from which the concretes in the respective series were made.

**Figure 9:**
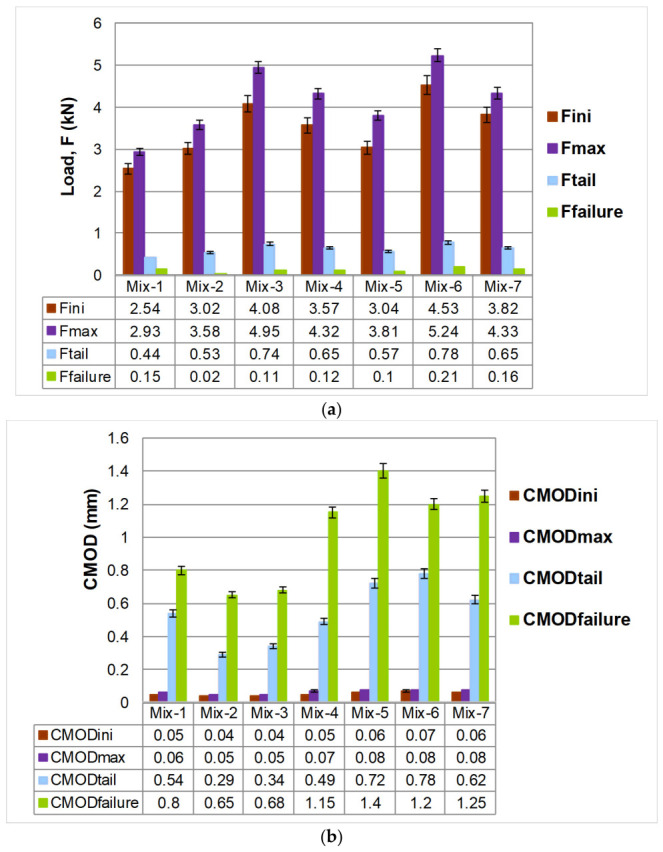
Distribution of significant parameters of *F*–*CMOD* envelope curves: (**a**) load, *F*; (**b**) *CMOD*.

Therefore, Figure 9 compiles and subsequently analyzes how the values of F and *CMOD* changed at the moment of

- Crack initiation (ini);
- Occurrence of the maximum force (max);
- Appearance of the tail on the *F–CMOD* curve (tail);
- Complete destruction of the specimen (failure).

By carefully analyzing both graphs compiled in Figure 9, it is evident that concretes with siliceous admixtures (especially the Mix-3 series) exhibit high values of *F*_ini_ and *F*_max_, but with the lowest values of *CMOD*_ini_ (0.04 mm) and *CMOD*_max_ (0.05 mm). Also, the values of *CMOD*_tail_ and *CMOD*_failure_ are at the lowest level in these concretes (0.29 mm and 0.65 mm for the Mix-2 series, and 0.34 mm and 0.68 mm for the Mix-3 series, respectively) (Figure 9b).

These results thus confirm the shape of the *F–CMOD* envelope curves obtained for concretes with the addition of NS alone and NS combined with SF. The sharp peaks on the envelope curves visible in Figure 7b,c and the rapid drop in force after cracking with a practically negligible tail section, combined with high *F*_ini_ and *F*_max_ values and very low *CMOD*_ini_, *CMOD*_max_, *CMOD*_tail_, and *CMOD*_failure_ values (Figure 7), indicate a high brittleness of the matrix reinforced with siliceous admixtures.

Conversely, concretes with the addition of FA in both ternary and quaternary systems (concrete series from Mix-4 to Mix-7) show a completely different tendency in the multi-stage material destruction process. The *CMOD*_tail_ and *CMOD*_failure_ values increase rapidly in these composites, reaching the highest values in the Mix-5 and Mix-7 series concretes (0.72 mm and 1.40 mm for the Mix-5 series, and 0.62 mm and 1.25 mm for the Mix-7 series, respectively) (Figure 9b). Additionally, the *F–CMOD* curves become “wider” with a clearly extended tail section of the graph. It was observed that for concretes with the highest amount of FA admixture, i.e., the Mix-5 and Mix-7 series, the *CMOD*_failure_ value was more than twice as high as the values obtained for concretes with siliceous admixtures (Figure 9b).

### 3.5. Two-Parameter Fracture Model (TPFM)

In order to quantify the observed phenomena in the fracture process of the subject concretes, which were discussed in detail in the preceding subsections, a two-parameter fracture mechanic model was used in accordance with the RILEM Draft Recommendations, TC 89-FMT [99]. Based on the recommendations contained in [96], the following fracture mechanic parameters were determined and then analyzed:

- Critical stress intensity factor ($K_{Ic}^{S}$);
- Critical crack tip opening displacement (*CTOD*_c_);
- Critical effective crack length (*a*_c_).

**Figure 10:**
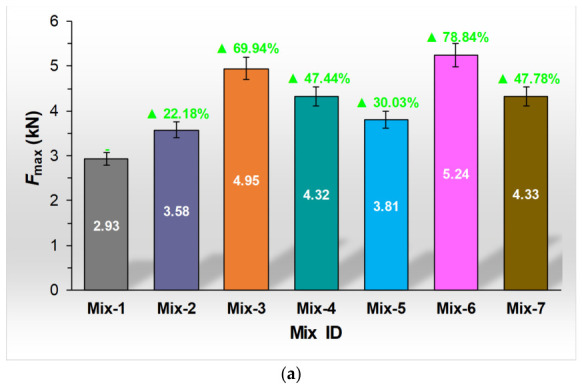

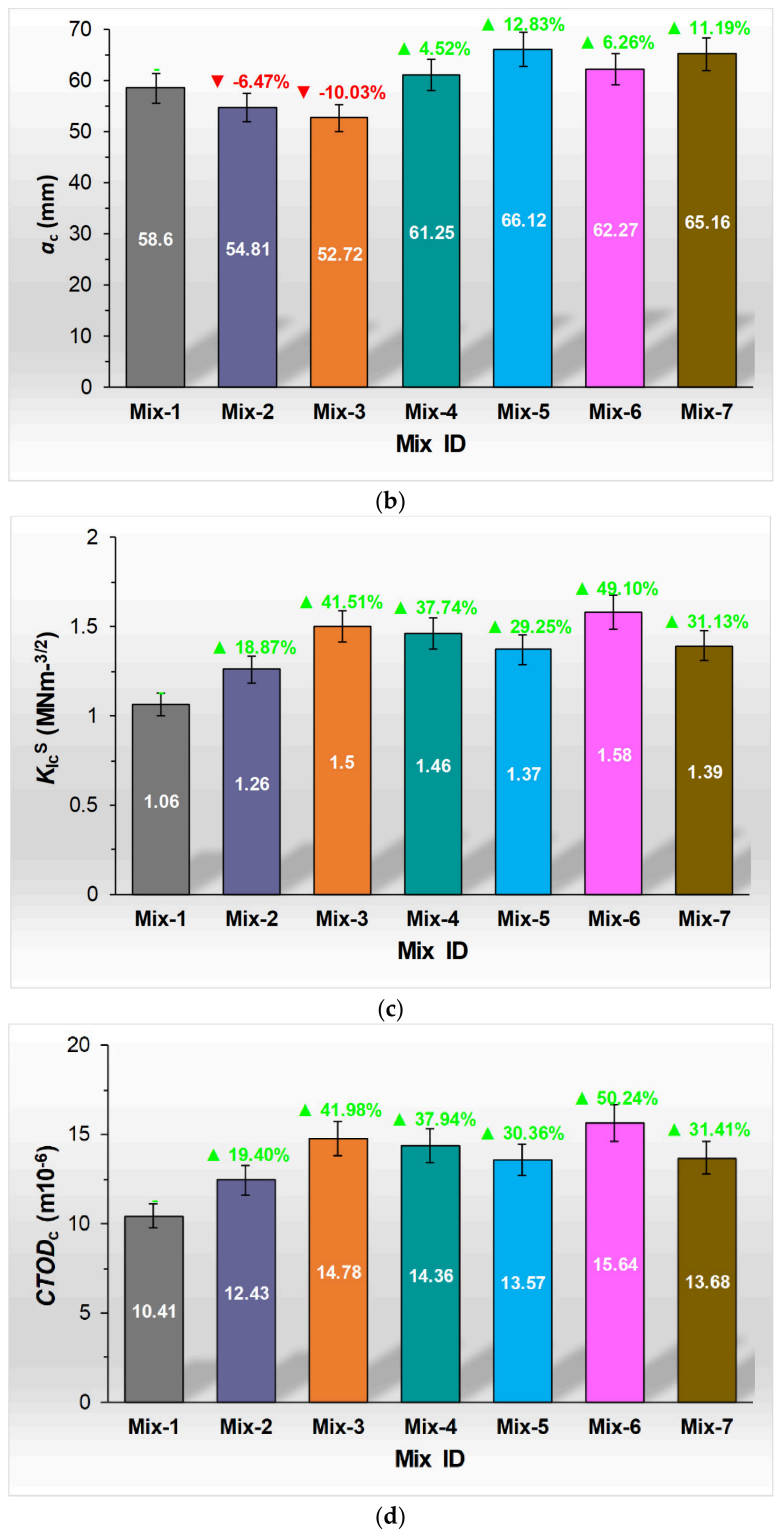
Fracture mechanic parameters of analyzed composites: (**a**) peak load, *F*_max_; (**b**) *a*_c_; (**c**) $K_{Ic}^{S}$; (**d**) *CTOD*_c_.

All essential equations used to determine the analyzed fracture mechanics parameters are compiled in the summary Table 4. Figure 10 and Table 6 present the average values of the analyzed fracture mechanic parameters. Additionally, to conduct a comprehensive analysis of the fracture process in composites in elastic terms, this figure also includes the mean values of the *F*_max_ forces.

Analyzing the data compiled in Figure 10 and Table 6, a clear convergence of the fracture toughness results in elastic terms with the values of the strength parameters of the tested concretes shown in Figure 7 must be noted. As could have been predicted, along with the increase in strength of individual concrete series, both the failure load Fmax and the other two significant fracture mechanics parameters, i.e., $K_{Ic}^{S}$ and *CTOD*_c_, also increased.

Within the set of other analyzed parameters, an interesting phenomenon is the change in the critical crack length *a*_c_ and the mutual *a*_c_/*a*_0_ proportions. The obtained results shed new light on the development of cracking in the subject composites. For the Mix-2 and Mix-3 series concretes, a decrease in this parameter was recorded by −6.47% and −10.03%, respectively, compared to the reference concrete. Such results clearly indicate an increase in the brittleness of these materials because the cracks become so sharp and the matrix so stiff that cracks propagate within a smaller effective failure zone. This is also evidenced by the lowered *a*_c_/*a*_0_ ratio compared to the reference concrete. In the Mix-2 and Mix-3 series concretes, it adopts the lowest values of all analyzed concretes, 9.62 and 5.44, respectively. This clearly demonstrates that these materials were highly sensitive to the appearance of initial cracks and tolerated their occurrence very poorly, and material failure occurred shortly after their emergence.

However, the initial drop in the *a*_c_ value for the Mix-3 series concrete, associated with the sudden cracking of this concrete, as shown in Figure 10b, is arrested in multi-component mixtures with the addition of FA. In Mix-5 and Mix-7, the ac value increases significantly, meaning that an extensive microcracking zone, the so-called Fracture Process Zone (FPZ), forms ahead of the main crack tip. This zone effectively dissipates fracture energy at the stage before cross-sectional separation occurs. The significantly higher values of the *a*_c_/*a*_0_ indicator, amounting to 32.24 and 30.32 for the Mix-5 and Mix-7 series concretes, respectively, also suggest that concretes modified with the combination of NS+SF+FA admixtures exhibited distinctly quasi-plastic behavior during the cracking process. In the concretes of these series, the crack could develop significantly before failure, while the material exhibited greater stress redistribution capacity and stability in crack propagation. Concretes with the addition of all types of SCMs possessed a greater “tolerance” for crack development, a more stable fracture pattern, and a higher share of microcracking prior to failure. This is confirmed by both the extended shapes of the *F–CMOD* curves shown in Figure 7.

Thanks to this, the fundamental fracture mechanic parameters, both $K_{Ic}^{S}$ and *CTOD*_c_, achieve the highest values for the Mix-6 series concrete. A distinct increase of approximately 50% in both material indicators was observed in this material. The obtained results thus confirm the highest load-bearing capacity of this series’ concrete regarding main crack propagation within the material structure.

Based on the analysis of the above fracture toughness results of concretes modified with pozzolanically active mineral admixtures in the form of SF and FA combined with a nano-admixture, which was NS, it can be stated that the combined application of all three SCMs acts beneficially on the concretes’ fracture toughness, as these admixtures create a synergistic effect at the micro- and nanoscale of the cement matrix. This synergy affects both the crack initiation stage and the crack propagation process. Based on the macroscopic results and literature evidence, it is hypothesized that the synergistic effect in the NS+SF+FA binder system consists of NS and SF compensating for the slow reactivity of FA, while FA mitigates the excessive brittleness of systems rich in SF and NS. This is very important because SF or NS alone often increase strength but simultaneously can increase the brittleness of the concrete. The addition of FA partially eliminates this effect. Therefore, quaternary concretes with the addition of NS, SF, and FA have higher *CMOD* values (especially in *CMOD*_tail_) (Figure 8). This, in turn, translates to higher values of both $K_{Ic}^{S}$ and *CTOD*_c_.

Therefore, based on both the fracture mechanic parameter results, and the ac values and *a*_c_/*a*_0_ proportions contained in Table 6, it can be unambiguously stated that concretes with the addition of NS and NS+SF are characterized by increased fracture toughness but a low tolerance to the occurrence of cracks and a sudden, brittle failure mode. On the other hand, concretes containing the combined pozzolanic additions of NS+SF+FA are characterized by both very high fracture toughness and high resistance to crack propagation. Concretes of this type are ductilized as a result of the presence of FA grains within the cement matrix structure. This, in turn, contributes to a stable and time-distributed failure character of these materials.

### 3.6. Energetic Analysis of the Beam Fracture Process—Studies of the Fracture Energy G_f_ Distribution

#### 3.6.1. Global Fracture Energy G_f_

**Figure 11:**
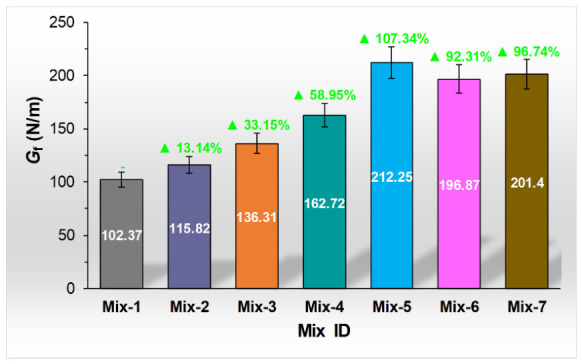
Total fracture energy, *G*_f_, of analyzed concretes.

In the presented research, the total fracture energy *G*_f_ was calculated based on the RILEM TC 50-FMC recommendations, outlined in study [101] and based on the work of fracture considering the division into specific zones of the cracking element defined in Section 2.4.2 and illustrated in Figure 6. The equation necessary to determine the total fracture energy *G*_f_ is provided in the comprehensive Table 4, while the average results obtained from these calculations are compiled in Figure 11.

Analyzing the total *G*_f_ results obtained for individual concretes, it is clearly evident that all composites modified with mineral additives were characterized by higher values of the analyzed parameter compared to the reference concrete. In the case of concretes with siliceous material additions, i.e., the Mix-2 and Mix-3 series, a small increase in total G_f_ of 13% and 33%, respectively, was observed compared to the reference concrete. Conversely, along with the modification of the binder composition by FA, a successive and pronounced increase in the G_f_ parameter value occurred. For the Mix-5, Mix-6, and Mix-7 series concretes, i.e., those with the highest FA content, an increase in the *G*_f_ level of 107%, 92%, and 96%, respectively, was observed.

The results compiled in Figure 11 prove that concretes with the addition of NS and NS+SF exhibit relatively low energy absorption capabilities during the cracking and fracture process. Therefore, such concretes, despite having quite high mechanical parameters (Figure 6) and relatively high fracture mechanics parameters (Figure 10), are unable to carry loads fully safely after the occurrence of initial material defects. Hence, the destruction of structures made from such materials, i.e., unable to adequately absorb the elastic energy originating from various types of external loads, may proceed in a sudden or catastrophic manner.

On the other hand, the high level of fracture energy observed in concretes with the addition of FA means that these materials can absorb a lot of energy before crack development and complete failure occur. The tests revealed record-breaking increases of over 100% and nearly 100% in the total *G*_f_ in the Mix-5, Mix-6, and Mix-7 series concretes. Such results suggest that the optimal combination of NS, SF, and FA is able to create a “tougher” material, capable of dissipating a tremendous amount of energy before complete destruction.

Therefore, concretes with the highest *G*_f_ values are less brittle and more ductile. Cracks in these materials develop slower, and after the appearance of the first crack, the concrete can still transfer a portion of the stresses. Structures made of such concretes better withstand the occurrence of dynamic and impact loads, as well as material fatigue, and exhibit greater resistance to catastrophic failure.

#### 3.6.2. Distribution of Fracture Energy Components

**Figure 12:**
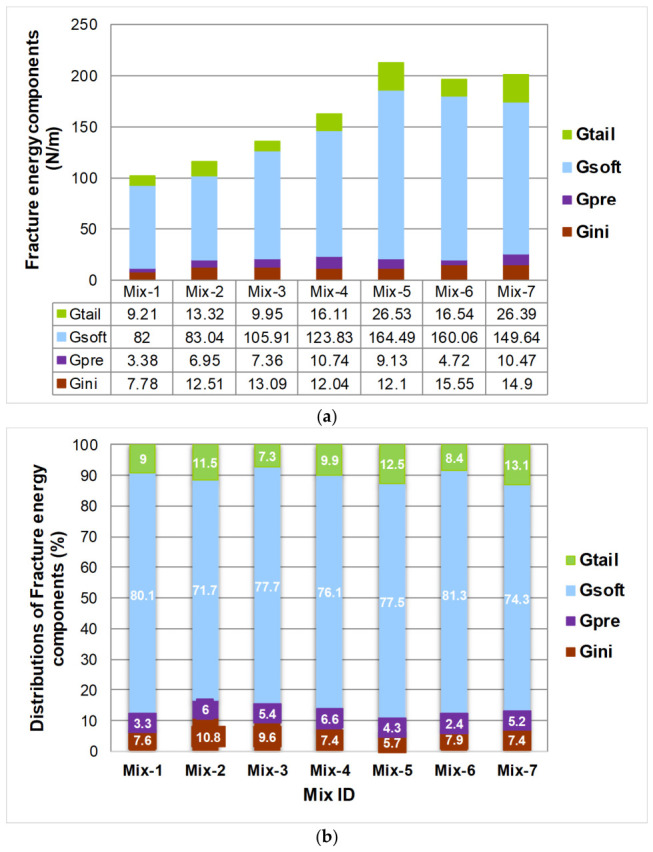
Energy absorption capacity of concretes: (**a**) absolute values of *G*_f_ components, (**b**) percentage distribution of the *G*_f_ components.

To conduct a detailed energetic analysis of the fracture process of the subject concretes, in addition to reporting the global *G*_f_ values, supplementary crucial configurations were selected. For this purpose, individual *G*_f_ components were specified, i.e., their absolute values (Figure 12a) and the percentage distribution of individual fractions of this parameter (Figure 12b). The consolidated results of these analyses are compiled in Figure 12. The detailed analysis of the *G*_f_ components (Figure 12) allowed for a “deeper” insight into the fracture process of individual concretes.

Partitioning *G*_f_ into four characteristic components revealed key differences in the behavior of individual concrete series during their cracking process. It should be noted that the dominant component in all concretes is *G*_tail_ (71–81%). However, the undisputed leader in energy absorption capacity proved to be the Mix-5 series concrete (*G*_f_ = 212.25 N/m). Such a distinct jump in the *G*_f_ level for this material compared to other concrete series stems primarily from the radical increase in the softening phase in this composite (*G*_soft_). Equally importantly, the longest “tail” of the *F–CMOD* curve was observed for the Mix-7 series concrete, which translated to the highest energy share in the final failure phase (*G*_tail_).

The obtained results thus show that a properly designed cement matrix consisting of 4 types of binder is capable of preventing sudden destruction of the structural element. Such a mechanism may be attributed to a decrease in the share of *G*_ini_ in the global *G*_f_ value in favor of a larger share of *G*_soft_ and *G*_tail_, which is a highly desirable feature from the perspective of structural safety.

Based on the results shown in Figure 12, the high share of *G*_tail_ in the Mix-5 (12.5%) and Mix-7 (13.1%) series concretes also draws attention. This confirms the thesis that FA in combination with NS and SF changes the cracking character from brittle to more ductile in the final failure phase.

### 3.7. Evaluation of the Brittleness and Deformability Index of Concretes

**Figure 13:**
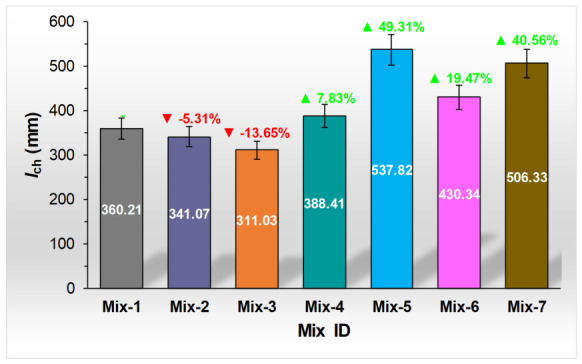
Characteristic length *l*_ch_ of the analyzed concretes.

The final stage of analyzing the performance of the concretes in question during their fracture process was the evaluation of the characteristic length *l*_ch_, determined from the formula proposed by Hillerborg and co-authors [102] and provided in Table 4. It should be noted that the *l*_ch_ parameter is also a measure of the material’s brittleness–ductility level and was selected as the ultimate indicator used to assess the impact of the applied material modification on these features of the analyzed concretes. Figure 13 presents the average *l*_ch_ values calculated on the basis of the previously determined constituent material parameters included in Equation (C2) (Table 4).

Based on the results shown in Figure 13, it can be stated that they confirm the earlier results of parameters describing the fracture characteristics of individual concretes. Similar to the ac values, the evaluation of *l*_ch_ also shows a decrease in the value of this parameter in the Mix-2 and Mix-3 series concretes. Lower *l*_ch_ values for these composites compared to the reference concrete, by 5.31% and 13.65%, respectively, indicate a dangerous shift in the material characteristic towards brittleness (a decrease in the “plastic zone” ahead of the crack front). These results thus correlate with the *a*_c_ values obtained for the concretes of these series, which also had lowered values compared to the Mix-1 series concrete (Figure 10b).

On the other hand, for the Mix-5, Mix-6, and Mix-7 series concretes, this parameter sharply increases by nearly 50%, almost 20%, and over 40%, respectively. Such results, combined with the highest observed crack opening displacements *CMOD*_tail_ and *CMOD*_failure_ (Figure 7b) obtained for these materials, prove that the synergistic action of NS, SF, and FA effectively neutralizes brittleness, transforming the concrete into a material with a quasi-ductile fracture character.

In the analysis of the *l*_ch_ parameter results obtained for all types of analyzed concretes, the phenomenon of a brittleness “trap” can be observed in the Mix-3 series concrete. It manifests itself in the fact that despite the very high material strength and fracture toughness (Figure 6 and Figure 10), this material exhibits a decrease in *l*_ch_ by 13.65%. This means that the addition of NS and SF alone causes the concrete to become more brittle, with a dangerous shift in this characteristic.

At the other end of the spectrum are composites containing FA. The participation of this waste in the cement matrix structure clearly improves ductility, significantly increasing the l_ch_ values in the Mix-5 and Mix-7 series concretes, which guarantees the design of safe structures.

From a practical engineering perspective, the findings of this study offer critical guidelines for structural concrete design and material selection. While the modern construction industry frequently prioritizes maximizing compressive strength through the intensive use of highly reactive siliceous additions (NS and SF), our results explicitly highlight the structural risks of such a unilateral approach—namely, a drastic increase in material brittleness and the potential for catastrophic, sudden failure. For structural elements subjected to dynamic loads, vibrations, or potential impact, such brittle behavior is highly undesirable. The practical consequence of our research is the recommendation to buffer high-strength siliceous matrices with delayed-action supplementary cementitious materials, such as FA. The developed quaternary binder formulations (particularly Mix-6 and Mix-7) define a technological ‘sweet spot’ for practitioners. By adopting these specific hybrid mixtures, structural engineers can simultaneously achieve high load-bearing capacity (compressive strengths exceeding 50 MPa) and a safe, quasi-ductile failure mode characterized by high post-peak energy absorption (*G*_tail_). Consequently, utilizing such multi-component matrices not only enhances the structural integrity and service life of the elements but also significantly improves the sustainability of the construction process by replacing up to 30% of Portland cement with industrial by-products.

Furthermore, regarding occupational health and safety (H&S) in real-life industrial applications, the potential inhalation risks associated with handling raw nano-silica powder can be completely mitigated by utilizing it in the form of colloidal liquid suspensions during concrete batching. Once the concrete hardens, the nanoparticles become permanently bound and chemically integrated within the stable C-S-H gel matrix, posing no environmental or health hazards during the structure’s service life.

## 4. Conclusions

The primary aim of this research was to comprehensively evaluate the synergistic effects of multi-component quaternary binder systems (combining ordinary Portland cement with fly ash, silica fume, and nano-silica) on the macro-mechanical fracture behavior, brittleness mitigation, and sub-stage energy dissipation of high-performance concrete. Based on the systematic experimental and analytical investigation conducted to achieve this goal, the following principal conclusions are drawn:

(1) Principal Scientific Contributions:
  - The four-stage fracture energy framework (*G*_ini_, *G*_pre_, *G*_soft_, and *G*_tail_) effectively maps the concrete failure process.
  - Pure highly reactive silica additions (NS and SF) increase pre-peak energy but cause severe post-peak embrittlement.
  - Quaternary hybrid blends successfully mitigate this brittleness, shifting the mechanism toward controlled energy dissipation.
(2) Practical Implications:
  - Relying solely on silica additions to maximize strength risks sudden, catastrophic structural failure.
  - The developed quaternary binders optimize the balance between high strength (>50 MPa) and necessary structural ductility.
  - Replacing up to 30% of Portland cement with industrial by-products significantly enhances concrete sustainability.
(3) Limitations of this Study:
  - The matrix interpretations are derived from macroscopic testing without direct microstructural evidence (SEM/XRD/MIP).
  - This study was limited to static loading conditions at a standardized curing age.
(4) Recommendations for Future Research:
  - Advanced microstructural imaging is needed to observe nanoscale crack-bridging.
  - Testing under dynamic, impact, and cyclic loading should be conducted.
  - Quantitative LCA and LCC analyses are required to evaluate long-term eco-efficiency.

## Figures and Tables

**Table 1:** The main composition of the OPC and SCMs used (mass %).

Material\Constituent	SiO_2_	Al_2_O_3_	CaO	MgO	SO_3_	Fe_2_O_3_	K_2_O	P_2_O_5_	TiO_2_	Ag_2_O
OPC	15.00	2.78	71.06	1.38	4.56	2.72	1.21	-	-	-
Class F FA	55.27	26.72	2.35	0.81	0.47	6.66	3.01	1.92	1.89	0.10
Non-condensed SF	91.90	0.71	0.31	1.14	0.45	2.54	1.53	0.63	0.01	0.07
Konasil K-200 NS	>99.8	-	-	-	-	-	-	-	-	-

**Table 2:** Physical properties of binders used.

Material\Parameter	Specific Gravity (g/cm^3^)	Blaine’s Fineness (m^2^/g)	Particle Size (μm)	Appearance
OPC	3.11	0.33	40	White Gray
Class F FA	2.14	0.35	30	Dark Gray
Non-condensed SF	2.21	1.40	11	Black
Konasil K-200 NS	1.10	200	0.012	White

**Table 3:** Mix proportions of concrete specimens (kg/m^3^) and the approach to OPC replacement by mass.

Mix ID	OPC	FA	SF	NS	Water	SP	Sand	Gravel
Mix-1	352	0	0	0	141	0	676	1205
Mix-2	299.2	0	0	17.6	141	6	676	1205
Mix-3	281.6	0	35.2	17.6	141	6	676	1205
Mix-4	281.6	52.8	0	17.6	141	6	676	1205
Mix-5	246.4	88	0	17.6	141	6	676	1205
Mix-6	281.6	17.6	35.2	17.6	141	6	676	1205
Mix-7	246.4	52.8	35.2	17.6	141	6	676	1205

**Table 4:** Summary of analytical formulas and geometric functions used for determining mechanical and fracture mechanics parameters.

Analyzed Parameter	Calculation Equation		Reference
(A) Basic strength properties			
Compressive strength (*f*_cm_)	$f_{cm}=\frac{F}{d^{2}}$	(1)	[97]
Splitting tensile strength (*f*_ctm_)	$f_{ctm}=\frac{2F}{{\pi d}^{2}}$	(2)	[98]
(B) Fracture mechanic parameters related to TPFM			
Young’s modulus (E)	$E=\frac{6Sa_{0}V_{1}\left(\alpha \right)}{\left(C_{i\;}d^{2}b\right)}$,	(3)	[99]
	where $V_{1}\left(\alpha \right)=0.76-2.28\alpha +3.78\alpha^{2}-2.04\alpha^{3}+\frac{0.66}{\left(1-\alpha^{2}\right)}\;,$ $\alpha =\frac{\left(a_{0}+HO\right)}{\left(d+HO\right)},$ $C_{i}-initial\;compliance\;based\;on\;F-CMOD\;curve,$ HO−caliper gauge holder thickness, *S*, *L*, *d*, *b*, a_0_—according to Figure 4.		
Critical effective crack length (*a*_c_)	$a_{c}=\frac{EC_{u}d^{2}b}{6SV_{1}\left(\alpha \right)}$,	(4)	[99]
	where $C_{u}-unloading\;compliance\;based\;on\;F-CMOD\;curve.$		
Critical stress intensity factor ($K_{Ic}^{S}$)	$K_{Ic}^{S}=3$(F_max + 0.5ω)$\frac{S{\left(\pi a_{c}\right)}^{1/2}F\left(\alpha_{1}\right)}{2d^{2}b}$,	(5)	[99]
	where $F\left(\alpha_{1}\right)=\frac{1.99-\alpha_{1}\left(1-\alpha_{1}\right)\left(2.15-3.93\alpha_{1}+2.7{\alpha_{1}}^{2}\right)}{\sqrt{\pi^{1/2}\left(1+2\alpha_{1}\right)}{\left(1-\alpha_{1}\right)}^{3/2}}$, $\alpha_{1}=\frac{a_{c}}{d}$, $\omega =\frac{W_{0}S}{L}$, $W_{0}-self\;weight\;of\;the\;beam.$		
Critical crack tip opening displacement (*CTOD*_c_)	$CTOD_{c}=\frac{6F_{\max}Sa_{c}V_{1}\left(\alpha \right)}{Ed^{2}b}{\left[{\left(1-\beta \right)}^{2}+\left(1.081-9\alpha \right)\left(\beta -\beta^{2}\right)\right]}^{1/2}$	(6)	[99]
	in which: $\alpha =\frac{a_{c}}{d}$,$\beta =\frac{a_{0}}{a_{c}}$.		
(C) Energetic and brittleness parameters			
Fracture energy	*G*_f_ = *G*_ini_ + *G*_pre_ + *G*_soft_ + *G*_tail_.	(7)	[101]
Characteristic length	$l_{ch}=\frac{EG_{f}}{f_{ctm}^{2}}.$	(8)	[102]

**Table 5:** Significant features of *F–CMOD* envelope curves.

Mix ID	The Analyzed Feature of the *F*–*CMOD* Envelope Curve					
	*F*_max_ (kN)	*CMOD*_max_ (mm)	*E* (GPa)	C_i_ (μm/kN)	C_u_ (μm/kN)	C_u_/C_i_ (-)
Mix-1	2.93	0.061	29.60	6.20	108.66	17.52
Mix-2	3.58	0.057	33.64	5.46	76.10	13.93
Mix-3	4.95	0.054	36.88	4.98	62.43	12.53
Mix-4	4.32	0.072	36.19	5.08	101.99	20.07
Mix-5	3.81	0.111	35.07	5.24	172.58	32.93
Mix-6	5.24	0.093	37.43	4.91	107.82	21.95
Mix-7	4.33	0.104	35.54	5.17	155.85	30.14

**Table 6:** Fracture mechanic parameters and brittleness indicators.

Mix ID	Category of Analyzed Parameters								
	Linear Elastic Fracture Mechanics				Ductility and Brittleness				
	$K_{Ic}^{S}$ (MNm^−3/2^)	*CTOD*_c_ (m10^−6^)	*a*_c_ (mm)	*a*_c_/*a*_0_ (-)	*G*_ini_ (N/m)	*G*_f_ (N/m)	*G*_ini_/*G*_f_ (-)	*l*_ch_ (mm)	*l*_ch_/*a*_c_ (-)
Mix-1	1.06	10.41	58.60	17.20	7.78	102.37	0.076	360.21	6.147
Mix-2	1.26	12.43	54.81	9.62	12.51	115.82	0.108	341.07	6.223
Mix-3	1.50	14.78	52.72	5.44	13.09	136.31	0.096	311.03	5.899
Mix-4	1.46	14.36	61.25	22.50	12.04	162.72	0.074	388.41	6.341
Mix-5	1.37	13.57	66.12	32.24	12.10	212.25	0.057	537.82	8.134
Mix-6	1.58	15.64	62.27	24.54	15.55	196.87	0.079	430.34	6.861
Mix-7	1.39	13.68	65.16	30.32	14.90	201.40	0.074	506.33	7.771

## Data Availability

The original contributions presented in this study are included in the article. Further inquiries can be directed to the corresponding author.
